# Anterior Cruciate Ligament Reconstruction Using the Tape‐Active Reconstruction System With Semitendinosus Graft: A Primary Load‐Bearing Construct

**DOI:** 10.1002/atn2.70028

**Published:** 2026-04-29

**Authors:** Ahmet Emin Okutan, Lokman Kehribar

**Affiliations:** ^1^ School of Medicine Department of Orthopaedic Surgery Samsun University Samsun Turkey; ^2^ School of Medicine Department of Orthopaedic Surgery Dokuz Eylül University İzmir Turkey

## Abstract

Anterior cruciate ligament reconstruction is a widely performed orthopaedic procedure; however, early graft elongation and loss of tension remain significant biomechanical challenges, particularly with the use of soft tissue autografts. In this technical note, the tape‐active reconstruction system is described as an augmentation technique in which a high‐strength suture tape functions as the primary load‐bearing element, while the semitendinosus tendon graft provides a biologic scaffold to facilitate osteointegration. This construct is designed to resist early elongation, preserve graft positioning, and enhance joint stability during the critical early phase of healing. The tape‐active reconstruction system represents a promising strategy for improving initial graft protection and optimizing long‐term outcomes in anterior cruciate ligament reconstruction.

## Graphical abstract

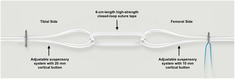

**VIDEO 1:** Surgical technique of anterior cruciate ligament reconstruction in a right knee using the Tape‐Active Reconstruction System (TARS) with a semitendinosus autograft. The technique includes graft preparation, femoral and tibial tunnel creation, suspensory cortical fixation, and final assessment of graft orientation and stability. Video content can be viewed at https://doi.org/10.1002/atn2.70028.

Anterior cruciate ligament (ACL) reconstruction is one of the most commonly performed orthopedic procedures, yet graft failure and persistent laxity remain ongoing challenges.^1^ Although autografts—particularly semitendinosus and gracilis tendons—are widely used due to their availability and favorable biomechanical properties, they are inherently viscoelastic, leading to undesirable elongation and stress relaxation over time.^2^ Several biomechanical studies have shown that, despite adequate intraoperative pretensioning or preconditioning, soft tissue grafts used in ACL reconstruction experience a substantial decrease in their initial loading force over time.^3^, ^4^, ^5^ Reported reductions range from 49% to 78% within the first 15 minutes to 4 hours after fixation, depending on graft type and timing of measurement.^4^, ^5^ More recently, a biomechanical study reported a median loss of 91% in graft force within 24 hours following tibial‐sided soft tissue interference screw fixation—regardless of screw diameter or length.^3^

Independent suture tape augmentation has been introduced as a strategy to enhance graft protection in ACL reconstruction, drawing on its favorable biomechanical properties—particularly its ability to function like a seatbelt by distributing forces and resisting elongation.^6^, ^7^ Based on this principle, the tape‐active reconstruction system (TARS) was developed to ensure a more stable and predictable mechanical environment during the critical early phase of healing. In the TARS construct, a high‐strength suture tape serves as the primary load‐bearing component, while the semitendinosus autograft functions solely as a non–load‐bearing scaffold to support osteointegration and biological maturation. This approach is intended to preserve graft positioning, prevent elongation, and ensure consistent joint stability during the early postoperative period.

## SURGICAL TECHNIQUE

A detailed video of the technique is shown in Video 1.

### Patient Positioning and Preparation

The patient is placed in the supine position on the operating table. After induction of regional anesthesia, a thorough knee examination is performed to assess ligamentous stability and range of motion. A well‐padded high‐thigh tourniquet is then applied to the operative leg.

### Diagnostic Arthroscopy

Standard anterolateral and anteromedial arthroscopic portals are established. A systematic diagnostic arthroscopy is then performed to assess the entire joint. Any concomitant intra‐articular pathologies are identified and addressed as necessary. ACL rupture is confirmed.

### Semitendinosus Harvest and Graft Preparation Using TARS

Only the semitendinosus tendon is harvested using a tendon stripper, while the gracilis tendon is typically preserved. The harvested graft is soaked in 1 g of vancomycin powder solution both before and after preparation to reduce the risk of infection.

**FIGURE 1:**
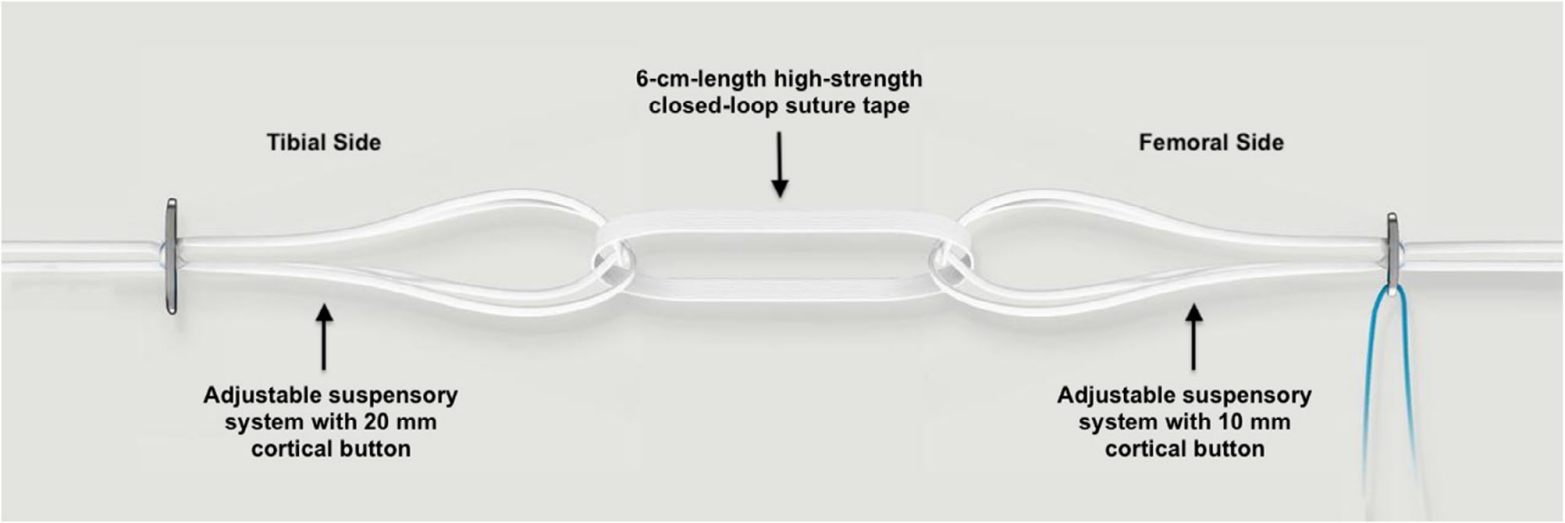
Schematic representation of the TARS. The TARS construct consists of 2 adjustable suspensory fixation devices connected by a 6‐cm‐long high‐strength closed‐loop suture tape. The femoral button measures 10 mm and the tibial button 20 mm, allowing precise cortical fixation and adjustable tensioning. This design replicates the native ACL configuration, with the suture tape acting as the primary load‐bearing element. (ACL, anterior cruciate ligament; TARS, tape‐active reconstruction system.)

**FIGURE 2:**
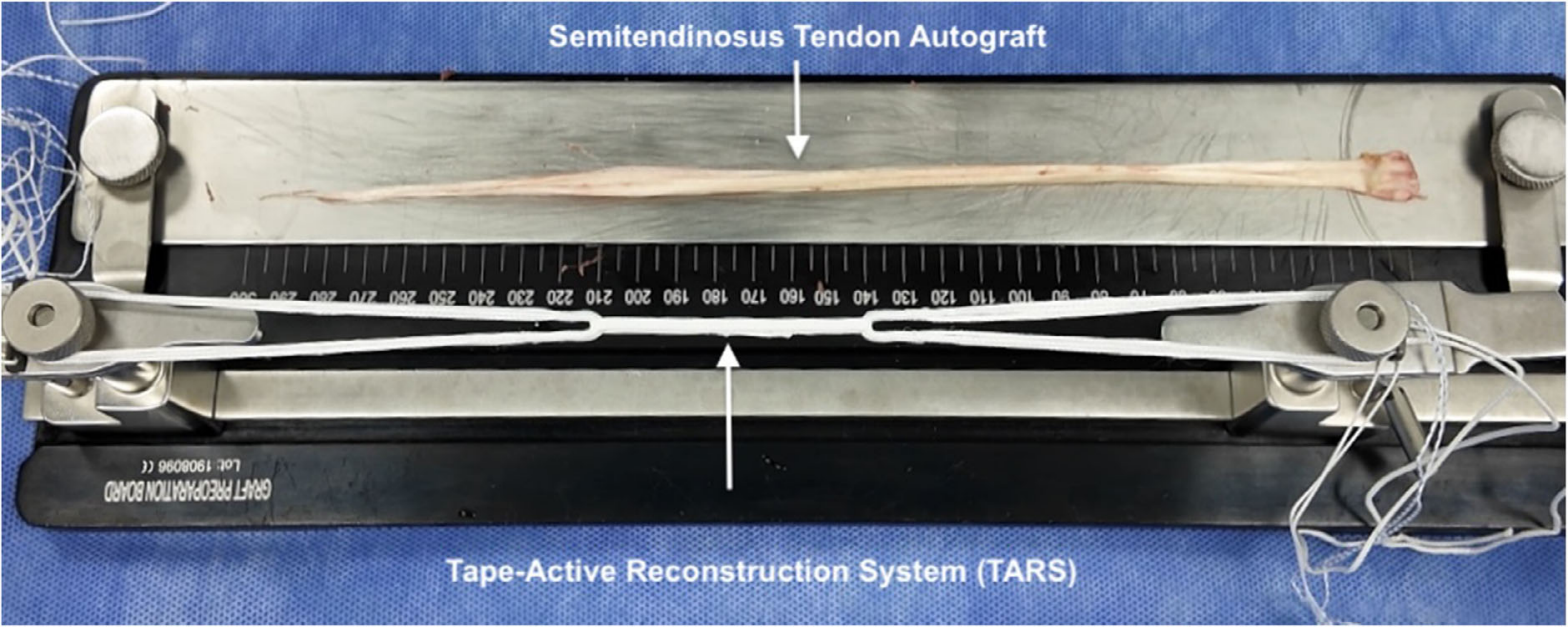
Intraoperative photograph of the semitendinosus autograft and TARS. (TARS, tape‐active reconstruction system.)

**FIGURE 3:**
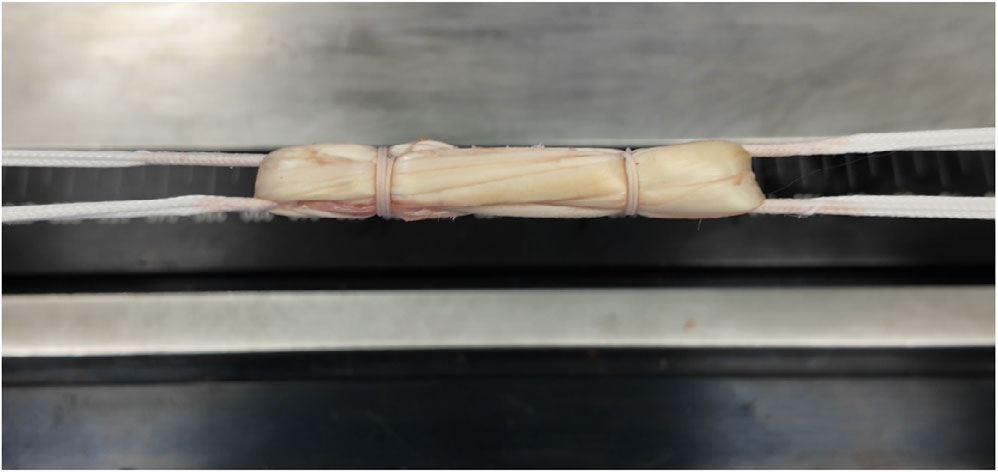
Prepared graft construct showing a four‐strand semitendinosus tendon wrapped around the central suture tape of the TARS. Circumferential FiberWire sutures secure all limbs of the graft and the central tape, creating a robust load‐bearing construct that combines mechanical stability with biological potential for osteointegration. (TARS, tape‐active reconstruction system.)

The TARS is composed of 2 adjustable suspensory fixation devices connected by a 6‐cm‐long high‐strength closed‐loop suture tape (Orthomed, Turkey) (Figure 1). Each device includes a cortical button—10 mm in width for the femoral side and 20 mm for the tibial side—allowing precise cortical fixation and adjustable tensioning. The 6‐cm tape length is designed to replicate the native ACL configuration, allocating approximately 3 cm intra‐articularly and 15 mm into both femoral and tibial sockets. A minimum graft length of 24 cm is required to achieve a complete quadrupled wrapping around the central tape; however, a length of 25 to 26 cm is preferred to provide adequate coverage and enable reliable fixation with sutures (Figure 2). The graft is folded longitudinally and wrapped evenly around the central suture tape to form a quadrupled configuration, with the tape positioned centrally along the axis of the construct. To secure the configuration, four No. 2 FiberWire sutures are applied in a circumferential fashion at either end. It is important that each limb of the graft is captured with this wrapping suture and the central suture tape. The final construct features the suture tape as the primary load‐bearing element, fully enclosed by the autograft, which provides a biological scaffold for osteointegration and graft maturation (Figure 3).

### Femoral and Tibial Tunnel Creation

**FIGURE 4:**
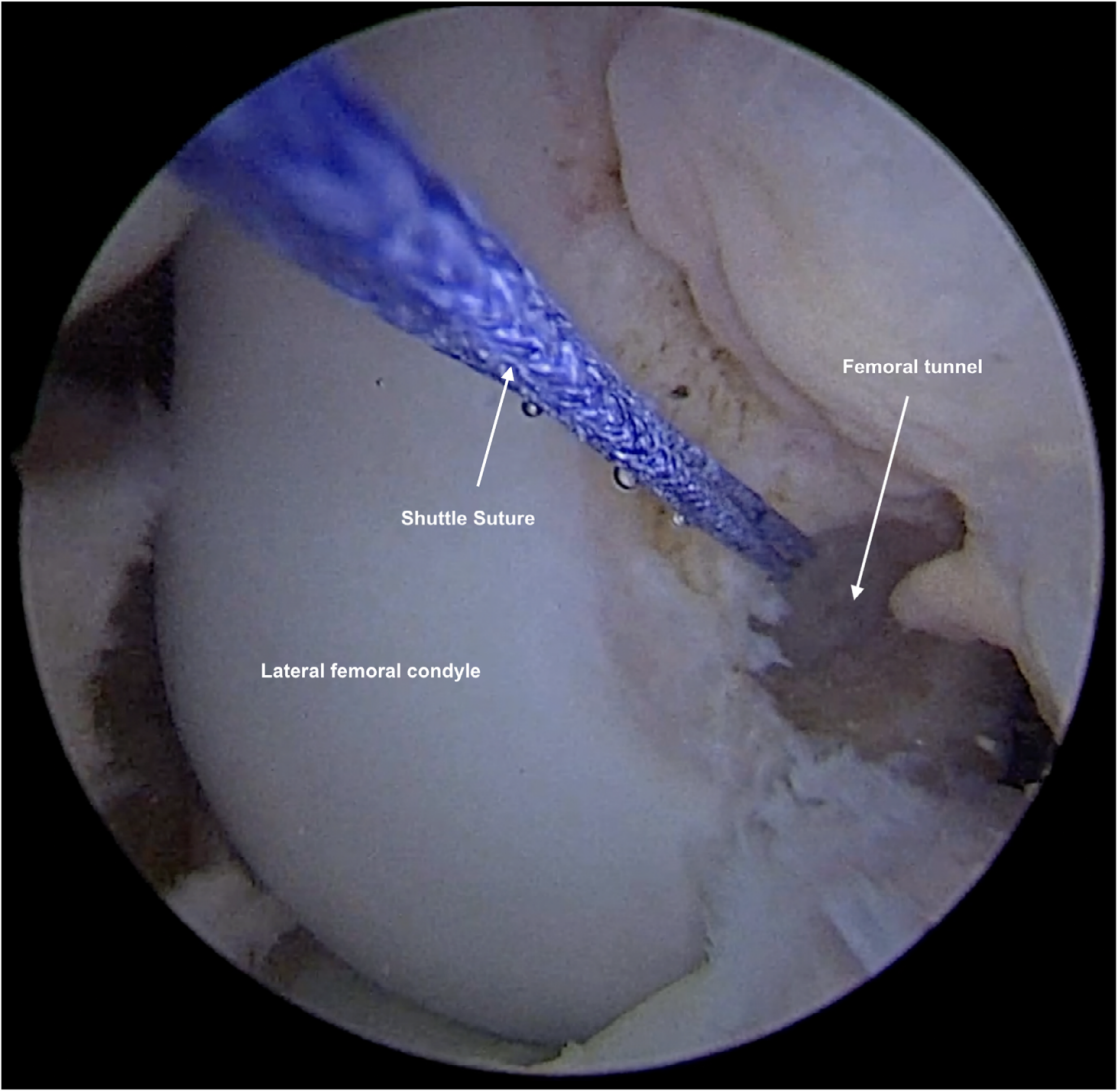
Arthroscopic view of the right knee through the anteromedial portal showing the positioning of the femoral tunnel. The patient in supine position and the knee flexed to 90°.

With the knee flexed to 110°, the anatomic femoral tunnel is first created via the anteromedial portal using a femoral aimer. A complete tunnel is established by advancing a 4.5‐mm drill over a guide pin. Subsequently, a 15‐mm femoral socket is reamed using a drill matching the graft diameter (Figure 4). For the tibial side, a full tunnel is prepared using an outside‐in technique with a tibial aimer set at an angle of 60° to 65°, targeting the central portion of the anatomic tibial footprint.

### Graft Placement and Fixation

**FIGURE 5:**
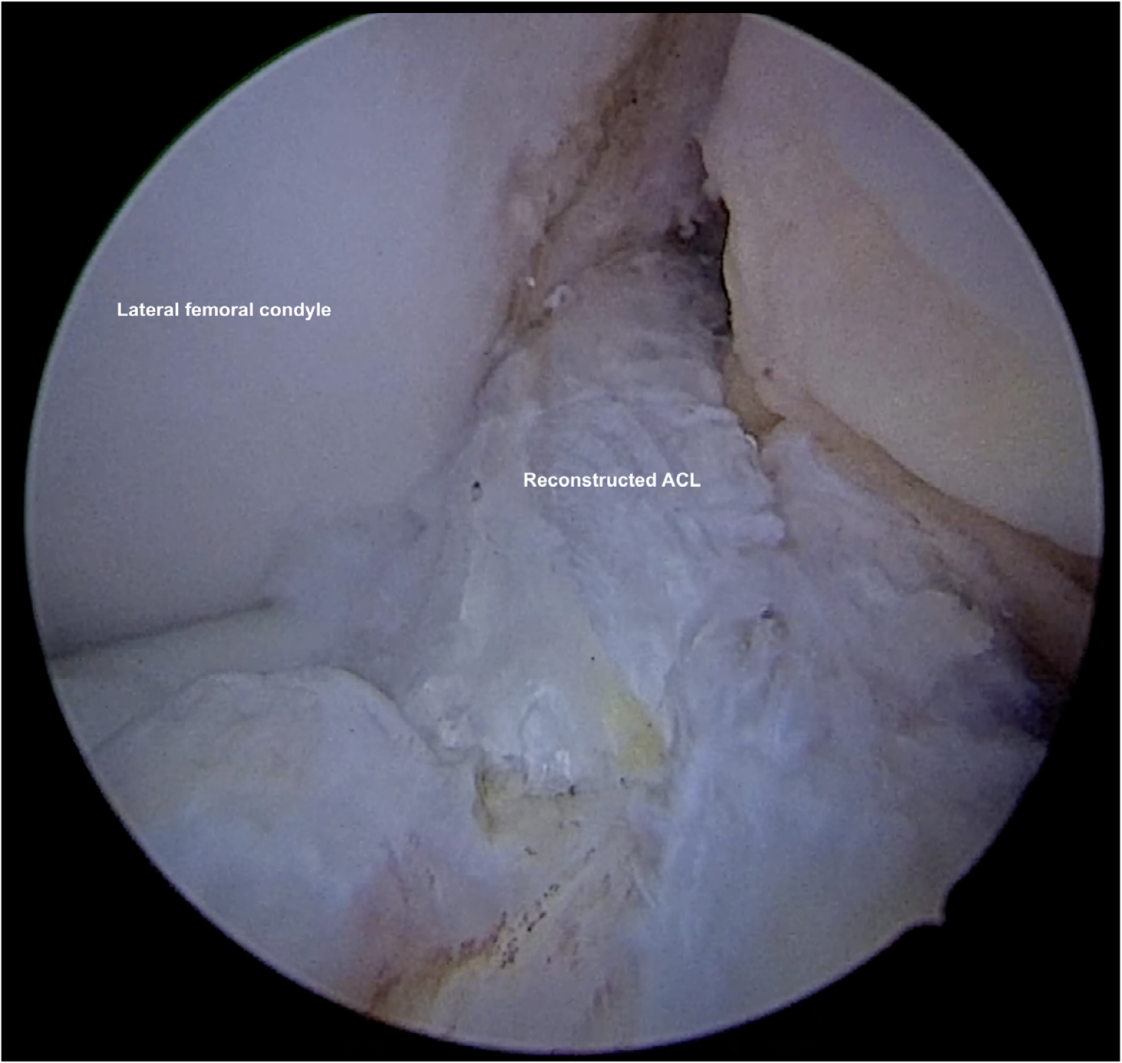
Arthroscopic view of the right knee through the anteromedial portal showing the final position of the quadrupled semitendinosus graft secured with the TARS. (TARS, tape‐active reconstruction system.)

The graft construct is delivered through the tibial tunnel and advanced into the femoral socket. Once the femoral suspensory button passes the femoral tunnel, it is flipped against the lateral femoral cortex to achieve cortical fixation. The tensioning sutures are alternately tensioned to draw the graft securely into the femoral socket. Subsequently, the tibial suspensory button is deployed, and the knee is brought into full hyperextension by holding the foot. Final tibial fixation is achieved by tightening the tensioning sutures on the tibial side. The knee is cycled several times to eliminate any slack in the construct, and both femoral and tibial tensioning sutures are retensioned in full hyperextension to ensure optimal graft fixation (Figure 5).

### Postoperative Rehabilitation

Full weight‐bearing and progressive range‐of‐motion exercises are encouraged from the early postoperative period. Initial rehabilitation focuses on achieving full knee extension and restoring quadriceps activation. Emphasis is placed on closed‐chain strengthening exercises to promote joint stability and neuromuscular control. As recovery advances, patients gradually progress to sport‐specific activities in accordance with standardized rehabilitation protocols.

## DISCUSSION

In recent years, suture tape augmentation of ACL reconstruction has emerged as a promising technique to enhance the mechanical performance of ACL grafts during the early phase of healing.^7^, ^8^, ^9^ By providing additional tensile resistance, especially in smaller‐diameter grafts, suture tape has been shown to reduce elongation, improve construct stiffness, and potentially lower graft failure rates during the vulnerable remodeling period.^6^ This load‐sharing mechanism—often referred to as the “seatbelt effect”—may protect the graft against early laxity, particularly in small‐diameter constructs that are prone to failure. However, current literature reveals no consensus regarding the clinical efficacy of suture tape augmentation in ACL reconstruction, with some systematic reviews reporting biomechanical benefits and potential improvements in graft healing, while others find insufficient evidence for superior clinical outcomes compared with standard techniques.^6^, ^10^

Nevertheless, concerns remain regarding the possibility of stress shielding, where excessive mechanical protection might reduce the biological stimuli necessary for effective graft maturation and tendon‐bone integration.^11^ While this remains a theoretical risk, clinical evidence directly supporting detrimental effects of stress shielding in ACL reconstruction is limited. Much of the concern originates from a histological animal study by Itoh et al.,^12^ which reported alterations such as decreased collagen fibril density, reduced collagen area, and differences in mineralization patterns at the graft‐bone interface under stress‐shielded conditions. However, this study did not include biomechanical outcome measures, limiting the extent to which its findings can be translated to clinical scenarios. While these results highlight the importance of maintaining appropriate mechanical loading, they do not conclusively show that suture tape augmentation compromises biological healing in ACL reconstruction.

The semitendinosus‐combined TARS technique was developed in light of this biomechanical‐biological balance. Unlike traditional augmentation methods where the tape shares load with the graft, TARS positions the high‐strength suture tape as the primary load‐bearing component, while the semitendinosus autograft is configured as a quadrupled, non–load‐bearing scaffold. The aim is to provide mechanical stability in the immediate postoperative period—when the graft is biologically weakest—without subjecting it to potentially deleterious forces. While this design effectively reduces early elongation risk, it does not entirely isolate the graft from physiological load or biological healing. The graft remains in contact with the bone tunnels and participates in healing processes such as osteointegration and synovialization. We summarize the pearls and pitfalls of our technique in Table 1 and the advantages and disadvantages in Table 2.

**TABLE 1:** Advantages and Disadvantages of the Semitendinosus‐Combined TARS Technique

Advantages
• Provides primary load‐bearing through high‐strength suture tape • Minimizes graft elongation and maintains joint stability in early healing • Enables accelerated rehabilitation protocols due to mechanical consistency • Preserves the biological presence of autograft to support osteointegration • Reduces graft harvesting morbidity by using only semitendinosus tendon

Disadvantages
• Potential risk of stress shielding, though evidence remains limited • Risk of joint overconstraint if the tape is improperly tensioned • Lack of long‐term clinical outcome data

TARS, tape‐active reconstruction system.

**TABLE 2:** Pearls and Pitfalls of the Semitendinosus‐Combined TARS Technique

Pearls
• Ensure semitendinosus graft length is at least 25‐26 cm to allow for full quadrupling and secure wrapping. • Maintain central alignment of the suture tape within the graft bundle. • Apply final tensioning in full knee extension after cycling the joint. • Perform independent fixation of both suspensory buttons, ensuring secure deployment.

Pitfalls
• Inadequate graft length (<24 cm) may compromise construct integrity and fixation. • Insufficient tensioning or improper knee positioning may lead to early graft laxity. • Risk of joint overconstraint if the tape is improperly tensioned.

TARS, tape‐active reconstruction system.

In conclusion, the semitendinosus‐combined TARS technique provides a reconstruction paradigm that prioritizes mechanical consistency during early rehabilitation while maintaining the biological advantages of a tendon graft. Although theoretical concerns about stress shielding remain, current data suggest that controlled mechanical environments—rather than full stress elimination—may support more predictable outcomes. Future research should focus on long‐term biological responses to the semitendinosus‐combined TARS constructs, including remodeling patterns, tunnel healing, and return‐to‐sport performance, to fully assess its clinical potential.

## DISCLOSURES

The authors (A.E.O., L.K.) declare that they have no known competing financial interests or personal relationships that could have appeared to influence the work reported in this paper.

## INFORMED CONSENT

Informed consent was obtained from the patient's to use the patient's information.

## CONSENT TO PARTICIPATE

The informed consent form, permission to participate in this clinical study, and the publication of the data of this study in any journal were obtained from a participant included in this study.
